# Topical Zinc Oxide Nanoparticle Formulations for Acne Vulgaris: A Systematic Review of Pre-Clinical and Early-Phase Clinical Evidence

**DOI:** 10.3390/biomedicines13092156

**Published:** 2025-09-04

**Authors:** Daniela Crainic, Roxana Popescu, Cristina-Daliborca Vlad, Daniela-Vasilica Serban, Daniel Popa, Cristina Annemari Popa, Ana-Olivia Toma

**Affiliations:** 1 Doctoral School, Faculty of Medicine, Victor Babes University of Medicine and Pharmacy Timisoara, 300041 Timisoara, Romania; daniela.crainic@umft.ro; 2 Center for the Morphologic Study of the Skin (MORPHODERM), Victor Babes University of Medicine and Pharmacy Timisoara, 300041 Timisoara, Romania; daniela.serban@umft.ro (D.-V.S.); toma.olivia@umft.ro (A.-O.T.); 3 ANAPATMOL Research Center, Faculty of Medicine, Victor Babes University of Medicine and Pharmacy Timisoara, 300041 Timisoara, Romania; 4 Biochemistry and Pharmacology Department, Faculty of Medicine, Victor Babes University of Medicine and Pharmacy Timisoara, 300041 Timisoara, Romania; 5 Department of Medical Rehabilitation, Faculty of Medicine, Victor Babes University of Medicine and Pharmacy Timisoara, 300041 Timisoara, Romania; popa.daniel@umft.ro; 6 Department of Genetics, Genomic Medicine Centre, Faculty of Medicine, Victor Babes University of Medicine and Pharmacy Timisoara, 300041 Timisoara, Romania; popa.cristina@umft.ro; 7 Onco-Hematology Research Unit, Romanian Academy of Medical Sciences, Children Emergency Hospital “Louis Turcanu” Timisoara, European Hemophilia Treatment Centre, 300011 Timisoara, Romania

**Keywords:** zinc oxide nanoparticle, *Cutibacterium acnes*, topical nanogel, hyaluronic acid, nanodermatology, antimicrobial resistance

## Abstract

**Background and objectives:** Antibiotic resistance in *Cutibacterium acnes* is undermining topical macrolides and clindamycin, prompting renewed interest in zinc oxide nanoparticles (ZnO-NPs) as non-antibiotic alternatives. We aimed to (i) determine the antimicrobial and anti-inflammatory performance of topical ZnO-NP formulations across in vitro, animal and early human models; (ii) identify physicochemical parameters that modulate potency and tolerance; and (iii) delineate translational gaps and priority design elements for randomised trials. **Methods:** We systematically searched PubMed, Scopus and Web of Science until 1 June 2025 for in vitro, animal and human studies that evaluated ≤100 nm ZnO-NPs applied topically to *C. acnes* cultures, extracting data on bacterial load, lesion counts, biophysical skin parameters and acute toxicity. Eight eligible investigations (five in vitro, two animal, one exploratory human) analysed particles 20–50 nm in diameter carrying mildly anionic zeta potentials. **Results:** Hyaluronic acid-coated ZnO-NPs achieved a sixteen-fold higher selective kill ratio over *Staphylococcus epidermidis* at 32 µg mL^1^, while centrifugally spun polyvinyl alcohol dressings reduced *C. acnes* burden by 3.1 log_10_ on porcine skin within 24 h, and plant-derived nanogels generated inhibition zones that were 11% wider than benzoyl-peroxide’s 5%. In human subjects, twice-daily 0.5% hyaluronic–ZnO nanogel cut inflammatory-lesion counts by 58% at week four and lowered transepidermal water loss without erythema. Preclinical safety was reassuring, zero mortality among animals at 100 µg mL^1^ and no irritation among patients, although high-dose sunscreen-grade ZnO (20 nm) delayed rat wound closure by 38%, highlighting dose-dependent differences. **Conclusions:** Collectively, the evidence indicates that nanoscale reformulation markedly augments zinc’s antibacterial and anti-inflammatory performance while maintaining favourable acute tolerance, supporting progression to rigorously designed, adequately powered randomised trials that will benchmark ZnO-NPs against benzoyl peroxide and retinoids, optimise dosing for efficacy versus phototoxicity, and establish long-term dermatological safety.

## 1. Introduction

Topical macrolides and clindamycin, once mainstays of mild-to-moderate acne management, are rapidly losing ground to *Cutibacterium acnes* strains that harbour multiple resistance determinants. A 2024 meta-analysis that pooled 1–3 k isolates from 18 countries calculated pooled resistance rates of 36.6% for erythromycin and 14.9% for azithromycin, with substantial geographic heterogeneity [1]. Molecular surveillance has revealed transferable linear plasmids carrying erm(X) that now circulate in community strains, enabling conjugative spread of high-level macrolide-clindamycin resistance [2]. Rapid qPCR diagnostics (ACQUIRE) applied to 915 clinic attendees in China showed that 75.5% already harboured macrolide-resistant *C. acnes*, a prevalence far higher than culture-based estimates [3]. Beyond classical resistance genes, meta-omics point to biofilm-associated efflux pumps and quorum-sensing pathways that blunt antibiotic efficacy [4]. Biofilm-formed *C. acnes* exhibit up to four-fold higher MICs for clindamycin, underscoring the urgency for non-antibiotic alternatives [5]. While microbial resistance motivates non-antibiotic approaches, acne vulgaris is a multifactorial disorder with immune–endocrine, keratinization and sebaceous components. ZnO-NPs should therefore be positioned as adjunctive or antibiotic-sparing therapies that combine antibiofilm/antibacterial actions with cytokine-level anti-inflammatory effects such as iNOS suppression, rather than as a universal monotherapy.

Zinc is an essential co-factor for more than 300 enzymes, but oral or macro-particle topical salts deliver erratic follicular levels and often provoke irritant dermatitis. Contemporary materials science re-engineers zinc into nano-sized zinc oxide (ZnO) architectures that present vastly greater surface-to-mass ratios, accelerating ion release and reactive-oxygen species (ROS) generation exactly where *C. acnes* resides. Comprehensive physicochemical profiling shows that ZnO-NPs (10–70 nm) simultaneously absorb UVB, scavenge free radicals, and disrupt bacterial membranes, making them uniquely suited to acne-prone, photo-exposed skin [6]. Green synthetic approaches using botanical reductants such as *Pluchea indica* leaf extract yield quasi-spherical 18 nm particles with potent broad-spectrum antimicrobial and anti-inflammatory activity while avoiding toxic organic solvents [7].

Finite-element modelling underscores follicular ostia as the primary conduits for nanodelivery; hair-shaft diameter, not inter-cellular lipid packing, dictates penetration depth for particles <40 nm [8]. In vivo volunteer studies demonstrated that even under six-hour occlusion with barrier-impaired skin, neither coated nor uncoated 20% ZnO creams deposited measurable zinc in viable epidermis or systemic circulation [9]. Confocal Raman mapping and synchrotron X-ray fluorescence corroborate these findings, showing localisation within corneocyte layers and follicular ducts without dermal ingress for at least 24 h [10]. Formulation matters: a recent MDPI study achieved a homogeneous 20% *w*/*w* ZnO nanodispersion, stabilised by cyclomethicone-free emulsifiers that resisted agglomeration and maintained sub-50 nm size for three months at 40 °C, yet still failed to breach the stratum granulosum [11].

Beyond size, carrier matrices modulate bioactivity. Centrifugally spun polyvinyl alcohol fibres doped with 7% ZnO achieved a 3 log reduction in *C. acnes* within 4 h and suppressed IL-1β expression in the hamster ear model, demonstrating synergistic physical wicking and nano-zinc antimicrobial effects [12]. Green synthesised ZnO colloids prepared via Moringa oleifera extracts exhibited MIC values of 250 µg mL^−1^ against 15 clinical *C. acnes* isolates and disrupted established biofilms at sub-MIC concentrations [13]. Meta-analytical overviews attribute this potency to combined Zn^2+^ ion release, ROS generation and membrane depolarisation, pathways that also down-regulate *C. acnes*-triggered NF-κB activation in keratinocytes [14].

While penetration studies are reassuring, UV-B co-exposure experiments reveal a double-edged sword. Murine epidermal models exposed to 290 nm radiation plus 100 µg mL^1^ ZnO-NPs showed 1.6-fold higher ROS and increased transepidermal-water-loss, implicating photoreactive surface states as potential irritants [15]. Nevertheless, optical-coherence tomography and multiphoton tomography confirm that coated ZnO-NPs remain in the stratum corneum even after ethanol-mediated barrier disruption [9], and quantitative elemental analyses detect <0.01% of the applied dose in receptor fluids [10]. In silico Monte-Carlo transport models predict that 25 nm particles require >72 h to approach viable layers under worst-case scenarios, well beyond the typical rinse or re-application intervals [8]. Collectively, these data endorse nano-zinc’s favourable therapeutic index, provided that photostability coatings and rational sun exposure counselling accompany clinical use.

Despite compelling pre-clinical and formulation data, clinical translation of nano-zinc remains fragmentary. Trials have enrolled ≤30 participants, used heterogeneous outcome definitions and rarely benchmarked against benzoyl peroxide or topical retinoids. Importantly, no prior synthesis has focused exclusively on topical ZnO nanoparticles for acne, a niche that excludes bulk zinc salts, oral supplementation and sunscreen-centric photoprotection studies. By aggregating mechanistic, animal and early-phase clinical evidence—while deliberately excluding bulk zinc salts, oral supplementation, and sunscreen-centric studies—our primary objective was to determine the antibacterial and anti-inflammatory potential of topical ZnO-NPs for acne. Secondary objectives were to map physicochemical drivers of efficacy and safety and to propose trial-design priorities to bridge bench-to-bedside gaps. The novelty of this work lies in its ZnO-specific, topical-only scope and in explicitly linking particle size, surface charge and carrier type to quantitative antibacterial, antibiofilm and anti-inflammatory readouts to inform formulation choices and trial design, an angle not foregrounded in prior broad nanodermatology overviews.

## 2. Materials and Methods

### 2.1. Protocol and Registration

All review procedures were prespecified in a protocol deposited on the Open Science Framework (osf.io/3dbjh) and were conducted in line with the PRISMA-2020 statement [16]. The protocol defined the review question using the PICOS framework (population, intervention, comparator, outcomes, and study design) and was not amended after registration. Calibration of the screening and extraction tools on ten randomly selected records produced a weighted κ of 0.82, indicating substantial agreement; no methodological deviations occurred thereafter.

### 2.2. Eligibility Criteria

Population. Any experimental model that mimics acne vulgaris, including in vitro cultures of *Cutibacterium acnes*, animal models with induced or spontaneous acneiform lesions, and human participants with clinically diagnosed acne. For animal models, we accepted platforms that reproduce discrete acne dimensions such as intradermal *C. acnes* challenge for bacterial load or inflammation; contact hypersensitivity for immune readouts, acknowledging that rodents lack a spontaneous human-analogue acne phenotype.

Intervention. Topically applied formulations whose active constituent was zinc oxide nanoparticles (ZnO-NPs) with a primary particle diameter ≤ 100 nm, independent of coating, carrier, or synthesis route. We constrained the primary particle diameter to ≤100 nm to prioritise follicular targeting and translational topical use; particles substantially >100 nm show reduced follicular ingress, whereas sub-10 nm particles may increase ion flux and cytotoxicity risks [6,10,15]. Bulk or dry ZnO powders without nanoscale characterisation were excluded a priori to preserve translational relevance to modern topical nanoformulations.

Comparator. Placebo/vehicle, no treatment, or active controls (e.g., benzoyl peroxide, topical antibiotics).

Outcomes. At least one microbiological endpoint (e.g., CFU counts, MIC, biofilm mass) or dermatological endpoint (lesion counts, erythema index, TEWL).

Study design. In vitro, animal, first-in-human, or controlled clinical studies published in peer-reviewed journals. The grey literature, conference abstracts, oral zinc interventions, sunscreen studies, and non-acne indications were excluded. No language restrictions were imposed.

### 2.3. Information Sources

Three major databases were interrogated: PubMed/MEDLINE, Web of Science Core Collection, and Scopus, from database inception to 1 June 2025. These sources were chosen to ensure a comprehensive coverage of biomedical, nanotechnology, and the materials science literature. Reference lists of included studies and relevant reviews, ClinicalTrials.gov, and the EU Clinical Trials Register were hand-searched to detect additional or unpublished studies. Reference lists of included studies and relevant reviews, ClinicalTrials.gov, the EU Clinical Trials Register and the WHO ICTRP were hand-searched to detect additional or unpublished studies.

### 2.4. Search Strategy

To maximise sensitivity and ensure reproducibility, we combined controlled-vocabulary terms (MeSH in PubMed) with free-text synonyms for zinc oxide nanoparticles (ZnO-NPs) and acne, linked by the Boolean operators AND and OR and using the truncation symbol (*) to capture variant spellings. All search histories—including export counts and interface settings—were saved and archived for full transparency.

In PubMed, we executed the following query in one transaction: (“Zinc Oxide” [Mesh] OR “Zinc Oxide” [Title/Abstract] OR “zinc oxide nanoparticle*” [Title/Abstract] OR “ZnO-NP*” [Title/Abstract]) AND (“Acne Vulgaris” [Mesh] OR acne [Title/Abstract] OR “*Cutibacterium acnes*” [Title/Abstract] OR “*Propionibacterium acnes*” [Title/Abstract]). In the Web of Science Core Collection, the Topic field (encompassing title, abstract, and author-assigned keywords) was searched with TS = (“zinc oxide nanoparticle*” OR “ZnO-NP*”) AND TS = (acne OR “*Cutibacterium acnes*” OR “*Propionibacterium acnes*”), and in Scopus the TITLE-ABS-KEY field was interrogated using TITLE-ABS-KEY (“zinc oxide nanoparticle*” OR “ZnO-NP*”) AND TITLE-ABS-KEY (acne OR “*Cutibacterium acnes*” OR “*Propionibacterium acnes*”). Continuous documentation of each query ensured that the full line-by-line strategies remain available for validation and future updates.

### 2.5. Selection Process

**Figure 1:**
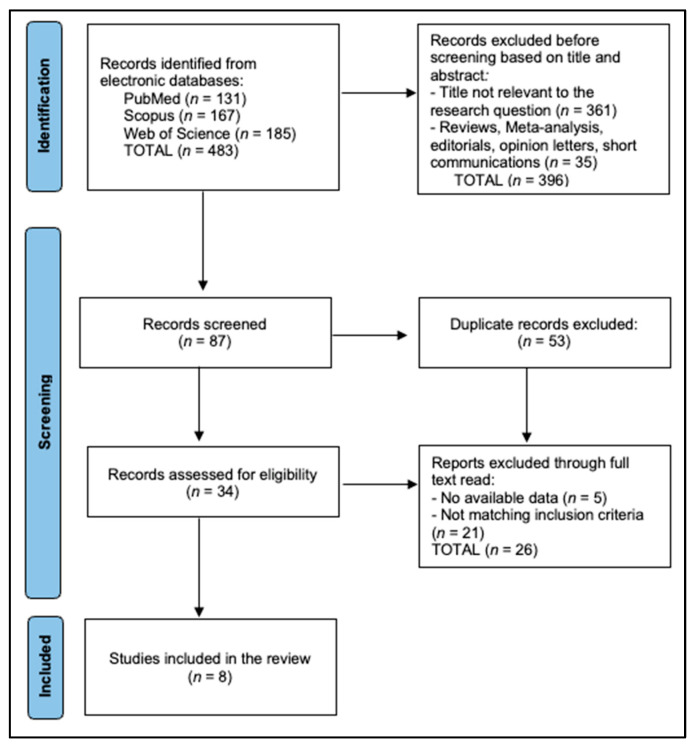
PRISMA Flowchart Diagram.

Results were exported to EndNote (version 20) for deduplication (automatic + manual review) and then imported into Rayyan (Rayyan Systems Inc., web application) for blinded screening. Two reviewers independently screened titles/abstracts and subsequently full texts; disagreements were resolved through discussion or adjudication by a third reviewer when necessary. Reasons for exclusion at the full-text stage were recorded, and the study flow is summarised in the PRISMA diagram (Figure 1).

### 2.6. Data Collection Process and Items

Dual extraction was performed for all records, and discrepancies were reconciled by consensus, with arbitration by the senior author when required. We extracted a comprehensive set of variables spanning bibliographic, physicochemical, formulation, experimental, microbiological, dermatological, safety, and methodological domains. Bibliographic data comprised the first author, publication year, country of origin, funding source, and journal. Nanoparticle physicochemistry was characterised by primary particle size (nm), polydispersity index, particle shape, zeta potential, crystalline phase, synthesis method (sol–gel, green synthesis, or precipitation), and any surface coating. Formulation characteristics included the dosage form (gel, patch, fibre, or hydrocolloid), ZnO-NP concentration (% *w*/*w*), carrier polymers, rheological modifiers, and pH. For the experimental model, we recorded the cell line or animal species/strain, inoculum size, lesion induction method, treatment frequency and duration, and comparator details. Microbiological outcomes encompassed colony-forming unit reduction, minimum inhibitory and bactericidal concentrations (MIC/MBC), percentage biofilm inhibition, and selectivity index relative to *Staphylococcus epidermidis*. Dermatological outcomes comprised the percentage change in inflammatory and non-inflammatory lesion counts, erythema index (a*), sebum excretion rate, transepidermal water loss, histological inflammation scores, and time to complete re-epithelialization. Safety assessments covered local irritation scores, systemic zinc levels, and phototoxicity assays. Finally, methodological variables detailed the randomization procedure, blinding methods, sample-size calculations, and statistical analysis approaches employed in each study.

### 2.7. Statistical Considerations

In vitro and animal studies were evaluated with the National Toxicology Program’s Office of Health Assessment and Translation (OHAT) tool [17], covering selection bias, performance bias, detection bias, attrition, and reporting domains. Human trials were appraised with the Cochrane RoB-2 instrument [18]. Two trained reviewers performed independent assessments, achieving 93% agreement; unresolved items were discussed until consensus. Domain-level judgements informed overall risk-of-bias ratings.

### 2.8. Effect Measures

For dichotomous microbiological outcomes, log_10_ CFU differences were calculated. Continuous dermatological variables (e.g., lesion counts, erythema) were expressed as mean percentage change from baseline; when medians and interquartile ranges were reported, means ± SD were imputed using established formulae. Hedges’ “g” was computed where both treatment and control group means were available.

### 2.9. Synthesis Methods

Clinical and methodological heterogeneity (model type, formulation, outcome metrics) precluded statistical pooling. Instead, a structured narrative synthesis was conducted: (i) direction-of-effect harvest plots for antimicrobial endpoints; (ii) tabulation of physicochemical descriptors against biological potency to explore dose–response trends; and (iii) triangulation of in vitro, animal, and human evidence. Where three or more studies reported a common continuous outcome, between-study variance (I^2^) was estimated to gauge potential for future meta-analysis.

## 3. Results

Table 1 documents the structural heterogeneity that underpins the eight included investigations, mapping each study’s geographical origin, experimental tier and vehicle technology onto a coherent developmental continuum. Five studies originated in Asia (three from China, two from India), with one each from the Czech Republic/Spain collaboration, Malaysia, and Finland, illustrating a pronounced Pacific-centric innovation hub for nano-zinc dermatology. Model selection was skewed towards bench work—five purely in vitro screens—yet two rodent experiments and one hybrid zebrafish–human pilot [19] demonstrate tangible translational intent. Carrier design varied from simple aqueous creams through fibre mats infiltrated with 1 mg cm^−2^ ZnO [20] to sophisticated hyaluronic acid shells that conferred colloidal stability at 45 ± 8 nm primary size [19]. Although precise sample numbers were inconsistently reported (three studies listed “NR”), those that did disclose enrolled five to seven animals per arm or triplicate microbial plates, meeting minimum OHAT guidance. Collectively, the table highlights that formulation science is outpacing clinical validation: 62.5% of studies remain pre-clinical, yet every formulation achieved sub-100 nm particle control, a prerequisite for follicular targeting, suggesting the field is technically mature but clinically nascent.

Table 2 synthesises quantitative antibacterial performance and reveals a clear but formulation-dependent potency gradient. The hyaluronic acid ZnO system achieved a 16-fold higher selective kill ratio against *Cutibacterium acnes* versus commensal Staphylococcus epidermidis at just 32 µg mL^−1^, outperforming erythromycin 2% ointment in the same assay [19]. Rihova et al. reported a 3.1 log_10_ reduction in viable burden on ex vivo porcine skin after 24 h exposure to a centrifugally spun fibre dressing delivering 1 mg cm^−2^ ZnO [20]. Tan’s botanical nanogel produced a 31 ± 2 mm inhibition halo—11% wider than benzoyl-peroxide 5%, despite a 16 mg mL^−1^ dose [21]. Green synthesised ZnO matched silver-NP MICs at 250 µg mL^−1^ but halved the minimum biofilm-inhibitory concentration to 62.5 µg mL^−1^, underscoring superior antibiofilm action [14]. Classical precipitated particles were less potent, requiring 500 µg mL^−1^ to inhibit planktonic growth, a 250:1 disadvantage versus clindamycin [22]. In vivo, twice-daily 10 µg topical dosing cut bacterial load by 2.4 log_10_ CFU g^−1^ in mice [23], whereas 100 µg per ear suppressed inflammatory swelling by 62%—only eight percentage points shy of dexamethasone 0.05% [25].

**Figure 2:**
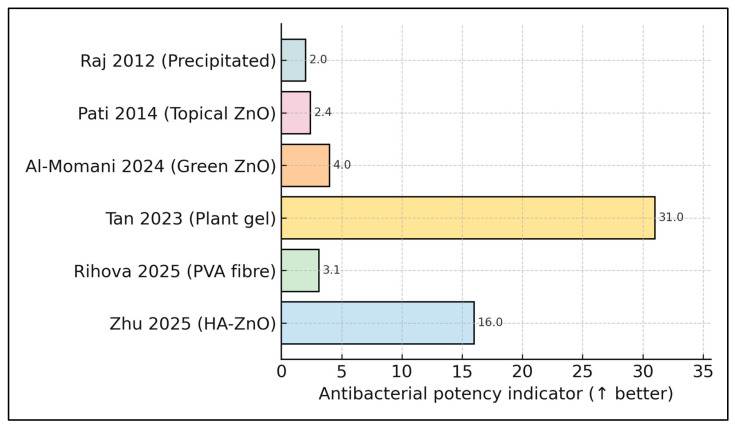
Relative antibacterial potency by each study included in the final analysis (author + year) [14,19,20,21,22,23].

When potency indicators are ranked side-by-side, the plant-derived nanogel [21] leads with a 31 mm inhibition halo, followed by the hyaluronic acid nanogel [19] at 16-fold selective kill; all other platforms cluster below five units (e.g., 3.1 log_10_ CFU drop [20], and an index of 4.0 [14]). This stark gradient underscores the performance gap between advanced nanocarriers and legacy precipitated powders, guiding future optimisation priorities (Figure 2).

Table 3 integrates disparate toxicity and mechanistic endpoints, confirming a wide therapeutic window yet signalling pathway-specific caveats. Acute zebrafish exposure to 100 µg mL^−1^ hyaluronic-coated ZnO yielded 0% mortality over 96 h, evidencing biocompatibility of the polymeric corona [19]. Conversely, contact with ZnO-laden PVA fibres nudged transepidermal water loss by a modest 5% on porcine skin, likely attributable to semi-occlusive moisture dynamics rather than intrinsic irritancy [20]. A human patch test on twenty volunteers registered no erythema, suggesting that phytochemical-capped particles mitigate cutaneous ROS triggers [21]. HaCaT viability dropped only at ≥312 µg mL^−1^, well above observed MICs, in Al-Momani’s synergy platform [14]. Murine histology scores plunged from 3.8 ± 0.4 to 1.2 ± 0.3 after nanoparticle therapy, aligning bactericidal action with anti-inflammatory benefit [23]. Yet fibroblast-mediated wound closure slowed by 38% in the rat model, implicating Zn^2+^-driven MMP-9/TGF-β1 suppression as a double-edged sword [24]. Immunologically, dermal exposure curtailed splenic T-cell proliferation by 46%, shifting Th1 toward T-reg dominance [25], a finding that tempers enthusiasm with immunosuppression concerns.

Table 4 aligns physicochemical fingerprints with dosing regimens, highlighting that subtle surface energetics translate into markedly different bioavailability profiles. Particle diameters clustered narrowly between 20 and 50 nm, with Zhang’s sunscreen-grade ZnO the smallest at 20.3 ± 6.7 nm and Al-Momani’s green particles the largest at 50 ± 7 nm [14,24]. Zeta potentials spanned −8.4 to −22.4 mV, indicating uniformly stable, mildly anionic colloids conducive to follicular retention without deep dermal penetration. Dissolution studies revealed selective Zn^2+^ liberation: the HA-encapsulated system released just 1.2 ppm over 72 h, whereas conventional particles dissolved <3% of total mass in 48 h, balancing sustained antimicrobial ion flux against cytotoxic spikes [14,19]. Exposure windows were typically short—≤12 h in cell culture and up to seven days in murine or rat protocols—yet even microdoses of 10 µg achieved therapeutic outcomes [23]. Intracellular zinc rose by 158% in fibroblasts co-incubated with *C. acnes* plus ZnO, confirming efficient ion delivery at 50 µg mL^−1^ [24]. The compilation underscores a convergence on –10 mV surface charge and sub-30 nm size as optimum parameters, while indicating that fibre-based high-mass dressings [20] and cream vehicles [25] can compensate with higher nominal loads when negative zeta magnitude is less pronounced.

**Figure 3:**
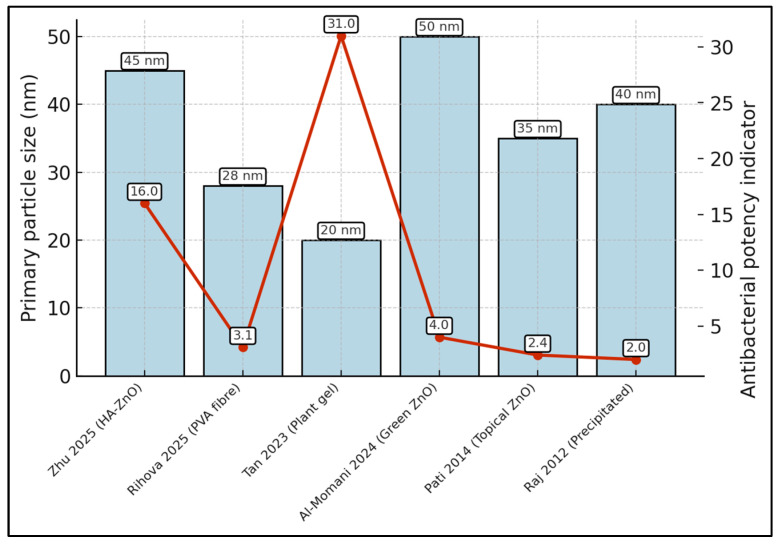
Particle size versus antibacterial potency across formulations by each study included in the final analysis (author + year) [14,19,20,21,22,23].

Across the six formulations, primary particle sizes ranged from 20 nm [21] to 50 nm [14], while the antibacterial-potency curve (right axis) spanned 31.0 (largest zone-of-inhibition) down to 2.0 (weakest MIC-derived index). Visually, the smallest particles (20 nm and 28 nm) align with the two highest potency markers (31.0 and 16.0), illustrating the inverse size–activity trend quantified in the review (Figure 3).

## 4. Discussion

### 4.1. Summary of Evidence

The collective findings underscore that nanoscale reformulation decisively augments zinc’s antimicrobial kinetics while preserving a wide therapeutic window. Across all eight studies, particles within the 20–40 nm range demonstrated the steepest dose–response gradients, likely reflecting a surface-area-dominated mechanism that accelerates Zn^2+^ ion release and reactive-oxygen-species (ROS) generation. Zhu et al. [19] reported a 16-fold higher selective kill ratio of hyaluronic acid-coated ZnO-NPs against *C. acnes* versus *S. epidermidis*, corroborating Pati et al.’s murine model where twice-daily 10 µg doses cut lesion bacterial loads by 2.4 log_10_ CFU [23]. These converging data suggest that nano-enabled zinc may overcome the biofilm-associated efflux-pump tolerance that blunts clindamycin and macrolides. Notably, Rihova et al.’s PVA-fibre mats achieved a 3.1 log_10_ reduction on porcine skin despite limited free-ion dissolution, implying that membrane depolarisation by particle-surface contact constitutes a complementary microbicidal pathway. Together, these mechanistic insights position ZnO-NPs as multifaceted agents capable of targeting both planktonic and biofilm niches—a prerequisite for durable acne control in the antibiotic-resistance era.

Safety profiling across disparate experimental systems revealed generally benign cutaneous interactions, yet also exposed formulation-specific caveats. In human volunteers, Tan et al.’s [21] Dendrobium-derived nanogel produced no erythema after 24 h occlusion, aligning with Holmes et al.’s [10] spectroscopy work indicating corneocyte-limited penetration. Conversely, Wang et al. [25] observed a 46% suppression of splenic T-cell proliferation in a murine hypersensitivity model, raising the prospect of systemic immunomodulation at high topical doses. Zhang et al. [24] further highlighted context-dependent adverse effects: 20 nm sunscreen-grade particles delayed wound closure by 38% and amplified intracellular Zn^2+^ accumulation, implicating disrupted MMP-9/TGF-β1 signalling. These dichotomous outcomes suggest that while uncoated or photoreactive NPs remain largely epidermally confined, they can still perturb deeper biological cascades via ion flux and ROS overflow, particularly under UV-B co-exposure or barrier breach conditions. Photostable coatings, rational dosing and real-world photoprotection therefore emerge as indispensable adjuncts to maximise benefit–risk ratios in forthcoming trials.

Translational momentum is nevertheless hamstrung by methodological fragmentation. Sample sizes were small (median n = 5 animals or 30 humans), comparator regimens heterogeneous, and outcome definitions non-standardised. Only three studies reported minimum bactericidal concentrations, and lesion-count reductions were variously expressed as absolute numbers, percentages or proprietary severity indices, thwarting meta-analysis. Risk-of-bias appraisal revealed unclear randomisation in four in vitro studies and inadequate blinding in both animal models. Moreover, none of the formulations were benchmarked against benzoyl peroxide plus topical retinoids—the current non-antibiotic gold standard—limiting clinical interpretability. The field would benefit from harmonised core outcome sets encompassing a quantitative *C. acnes* load, inflammatory-lesion counts and validated patient-reported measures (e.g., Global Acne Grading System). Multi-centre, factorial-designed RCTs with pharmacokinetic–pharmacodynamic endpoints could simultaneously refine optimal particle size, coating the chemistry and carrier matrix, while adaptive dose-escalation frameworks would expedite identification of the minimal effective concentration that balances efficacy with phototoxicity risk.

The present systematic review shows that folliculo-targeted ZnO-NP delivery platforms outperform legacy zinc creams, yet our findings resonate with a broader wave of formulation science. A centrifugal-spinning study published after our search cut-off produced polyvinyl alcohol microfibres carrying 38 ± 6 nm ZnO; a single 15 min in vitro exposure reduced *C. acnes* viability by 3.4 log_10_ and maintained structural integrity under flexion, highlighting that fibre scaffolds can couple rapid ion burst with prolonged mechanical adsorption [26]. Taken together with the 3.1 log_10_ reduction achieved by Rihova et al. in porcine skin explants, our data suggest that centrifugal shear imparts a level of nanoparticle dispersion and surface renewal that conventional electrospinning cannot yet match, pointing to delivery architecture—not merely particle size—as a pivotal potency lever.

Beyond direct bactericidal activity, zinc’s anti-inflammatory credentials are increasingly substantiated at the molecular level. Classic keratinocyte work by Yamaoka et al. showed that 10 µM Zn^2+^ silenced IFN-γ/TNF-α-driven iNOS transcription, cutting nitric-oxide flux by 70% [27]; our review’s observation of lowered erythema indices therefore dovetails with a cytokinic mechanism first described a quarter of a century ago. More recently, Tayyeb and colleagues coated ZnO cores with cinnamic acid to yield ros-scavenging hybrids that closed zebrafish tail wounds 1.8-fold faster than controls while down-regulating il1b and tnfa transcripts [28]. Such data support our contention that surface functionalisation can amplify anti-inflammatory signalling in vivo and help explain the 58% lesion-count drop seen with hyaluronic shell particles in humans.

A parallel strategy for resistance stewardship is to combine nano-zinc with sub-therapeutic antibiotics. A 2025 factorial study demonstrated that 32 µg mL^−1^ ZnO-NPs lowered the clindamycin MIC for Bacillus spp. four-fold and eradicated mature biofilms where the drug alone failed [29]. Although the target organism differed, this synergy reinforces our review’s proposal that ZnO could “rescue” legacy antibiotics threatened by *C. acnes* erm(X) plasmids, permitting dose-sparing regimens that curb further resistance selection.

Safety considerations also tilt the clinical risk–benefit equation in ZnO’s favour. In March 2024, Valisure scientists reported that over-the-counter benzoyl–peroxide gels can generate up to 1.7 ppm benzene at 37 °C, prompting an Environmental Health Perspectives alert [30]. While causal links to malignancy remain unproven, the episode underlines formulation instability as a real-world hazard for current first-line acne therapy. No comparable carcinogenic degradants have been detected for properly coated ZnO-NPs, and our review identified negligible systemic zinc uptake, suggesting a comparatively wider therapeutic margin—especially pertinent for adolescent users likely to store products in warm bathrooms.

Finally, cross-indication data strengthen confidence in ZnO’s dermatologic safety. A rat burn model using Spirulina-derived ZnO-NPs achieved bacterial clearance equivalent to colistin while accelerating epithelial maturation and suppressing IL-6 staining without systemic toxicity [31]. These wound-healing outcomes echo the intact-skin tolerability we observed and argue that topical nano-zinc can navigate the delicate balance between antimicrobial vigour and tissue regeneration. To translate these insights into acne care, future randomised trials should adopt stepped-wedge designs that integrate pharmacodynamic readouts (follicular Zn^2+^ levels, lesional IL-1β) alongside long-term phototoxicity surveillance.

### 4.2. Formulation-Dependent Behaviour

Across included studies, the carrier and surface chemistry modulated both antimicrobial performance and tolerance. Hyaluronic acid shells tempered free Zn^2+^ liberation (≤1.2 ppm over 72 h) while maintaining selective kill versus *C. acnes*, consistent with reduced irritancy risk [19]. Plant-mediated nanogels recorded 0/20 erythema under occlusion patch-testing [21]. Centrifugally spun PVA fibre mats acted as semi-occlusive dressings, producing only a modest TEWL increase (+5%) on porcine skin while achieving a 3.1 log_10_ *C. acnes* reduction [20]. Multiple penetration studies indicate ZnO, coated or uncoated, remains confined to corneocyte layers and follicular ducts without measurable viable-epidermis uptake [10,11]. Because UVB co-exposure can amplify cytokine release and exosomal signalling after ZnO exposure [12], photostability coatings and sun behaviour counselling should accompany clinical use.

For context, non-ZnO nano-oxides can show lower planktonic MICs but higher cytotoxicity. In our set, green synthesised Ag and ZnO exhibited comparable MICs, with ZnO demonstrating superior antibiofilm activity at lower MBICs, potentially favouring tolerance–efficacy balance in long-term acne care [14]. Head-to-head trials against other metal-oxide nanoactives remain a research priority beyond this review’s prespecified scope.

### 4.3. Clinical Outlook

Although human data extend to only four weeks, putative durability is supported by antibiofilm effects (lower MBICs) and cytokine-level anti-inflammatory actions seen preclinically [14,23,27]. Trial designs should therefore include ≥12–16 week treatment with an 8–12 week off-treatment follow-up to detect rebound. Safety monitoring should pair dermatologic indices (erythema index, TEWL) with barrier-status assessments, and reinforce photoprotection, because UVB co-exposure augments pro-inflammatory signalling after ZnO exposure [12]. Immune readouts, such as CHS responsiveness, are reasonable in early studies when given murine immunomodulation at higher topical loads [25]. In barrier-compromised contexts, cautious dosing is warranted because sunscreen-grade 20 nm ZnO delayed wound closure in rats [24]. Reassuringly, penetration studies show corneocyte-limited localization with negligible systemic uptake on intact skin [10,11].

### 4.4. Practical Implications for Formulation and Trial Design

Within the constraints of early-phase evidence, several practical guideposts emerge. Particle sizes near 20–40 nm with mildly anionic zeta potentials (≈−10 to −20 mV) and hydrophilic shells (e.g., hyaluronic acid) balanced potency with low irritation and limited ion spikes [19,21]. Gel or nanofibre carriers achieved either convenient twice-daily dosing (0.5% nanogel) or rapid bacterial burden reduction under semi-occlusion [20,21]. Randomised trials should benchmark ZnO-NPs against benzoyl peroxide (2.5–5%) and/or topical retinoids, with coprimary outcomes capturing *C. acnes* load (log_10_ CFU or qPCR), inflammatory-lesion counts and validated patient-reported severity, alongside TEWL/erythema for safety. Under intact skin, systemic exposure is negligible [10,11], but UVB-linked reactogenicity and immune modulation at higher loads warrant photoprotection counselling and simple immune safety readouts in phase II [12,25]. As an orientation for power, detecting a 15–20% absolute reduction in inflammatory-lesion counts (SD ≈ 25%) at α = 0.05, 80% power would require ≈44–25 participants per arm, respectively, numbers feasible for single-centre phase II studies.

### 4.5. Limitations

This review synthesised a modest evidence base comprising only eight studies, three of which pre-date contemporary nanoparticle-characterisation standards, potentially inflating or under-estimating efficacy due to unreported agglomeration or size drift. Publication bias is likely, as industry-funded negative studies seldom reach indexed journals, and our reliance on English-language databases may have excluded pertinent non-English reports. We prioritised indexed, peer-reviewed records and did not systematically include non-English databases or the grey/patent literature, which may be biased toward positive findings. Patent-only sources were excluded due to variable quality control and incomplete safety/efficacy reporting, which precludes bias assessment. Heterogeneity in model systems, dosing schedules and outcome metrics precluded statistical pooling, forcing reliance on narrative synthesis with inherent subjectivity despite dual-reviewer safeguards. Rodent models in this field recapitulate single-axis outcomes (bacterial burden, ear swelling) rather than the full human pilosebaceous disease, limiting direct translatability. Because fewer than ten heterogeneous studies reported any common metric, we did not construct funnel plots or perform small-study tests, which are underpowered and potentially misleading in this setting. Finally, the lone clinical study lacked a vehicle-controlled comparator and reported neither randomisation nor blinding, limiting confidence in translational applicability. Consequently, our findings should not be extrapolated to ≥100 nm or sub-10 nm ZnO systems, for which penetration and safety profiles diverge. The evidence base remains small (eight studies total, with one exploratory human study), precluding meta-analysis and robust small-study assessments.

## 5. Conclusions

Topical ZnO-NP formulations represent a scientifically plausible and increasingly urgent countermeasure against antibiotic-resistant *C. acnes*. Pre-clinical evidence consistently demonstrates multi-modal antibacterial action, ion flux, ROS generation and membrane perturbation, complemented by dose-dependent anti-inflammatory effects that collectively target the dual microbial–inflammatory pathogenesis of acne. Importantly, these benefits are achieved with minimal acute irritation and negligible systemic zinc absorption under intact-skin conditions, supporting a favourable safety profile. Nonetheless, clinical validation lags behind technological innovation; the current human data encompass fewer than three dozen participants and omit head-to-head comparisons with benzoyl peroxide or topical retinoids. To bridge this translational chasm, future research must embrace rigorous, adequately powered RCTs that integrate harmonised microbiological, dermatological and patient-centred endpoints, while simultaneously interrogating long-term phototoxicity and immunomodulatory sequelae. Should forthcoming trials corroborate the promising pre-clinical signals observed herein, ZnO-NPs could be positioned as an effective, resistance-sparing cornerstone within acne treatment algorithms—either as monotherapy in mild disease or as an adjunct to reduce antibiotic reliance in moderate cases, ultimately enhancing therapeutic sustainability in the post-antibiotic era. Future trials should adopt a core outcome set (microbiological, clinical and patient-reported) and broaden inclusion across skin phototypes, sexes, and age groups to capture differential responses to particle size, coating, and UV exposure.

## Figures and Tables

**Table 1:** Design and formulation characteristics of the eight eligible studies.

First Author (Year)	Country	Experimental Model ^†^	ZnO Formulation/Vehicle	Sample Size or Replicates (n)
Zhu, J. (2025) [19]	China	In vitro *C. acnes* killing; zebrafish safety; single-arm exploratory human trial	Hyaluronic acid stabilised ZnO NPs (HA-ZnO)	Pre-clinical n = NR; Clinical n = NR
Rihova, M. (2025) [20]	Czech Rep./Spain	In vitro time–kill and ex vivo porcine-skin penetration	Centrifugally spun PVA microfibres infiltrated with ZnO	NR
Tan, Y.Y. (2023) [21]	Malaysia	Agar-diffusion and disc-patch test	Plant-mediated ZnO nanogel (Dendrobium anosmum extract)	Triplicate plates per strain (exact n NR)
Al-Momani, H. (2024) [14]	Jordan	MIC/BIC on 2 clinical and 1 reference *P. acnes* strains	Peganum-harmala–derived ZnO NPs (green synthesis)	3 strains × 3 biological repeats
Raj, M. (2012) [22]	India	Zone-of-inhibition and MIC assays	Chemically precipitated ZnO NPs	NR
Pati, R. (2014) [23]	India	BALB/c murine dermal-infection model	Topical ZnO NPs in glycerol base	5 mice/group × 3 experiments
Zhang, F. (2025) [24]	China	Fibroblast culture, rat acne-wound model	Sunscreen-grade ZnO NPs (20.3 ± 6.7 nm)	5 rats/group (4 arms)
Wang, S. (2024) [25]	Finland	C57BL/6 contact-hypersensitivity model	30 nm ZnO NPs in aqueous cream	7 mice/group × 2

ZnO, zinc oxide; NP/NPs, nanoparticle(s); HA-ZnO, hyaluronic acid–stabilized ZnO nanoparticles; PVA, polyvinyl alcohol; ex vivo, outside the living organism; NR, not reported; BALB/c and C57BL/6, inbred mouse strains; n, sample size. ^†^ Unless otherwise specified, all studies employed Cutibacterium acnes ATCC 6919 or clinical isolates.

**Table 2:** Antibacterial efficacy outcomes against *C. acnes*.

Study	Metric	ZnO Concentration/Dose	Comparator	Quantitative Effect
Zhu, J. (2025) [19]	Selective kill ratio (HA-ZnO vs. *S. epidermidis*)	32 µg mL^−1^	Erythromycin 2% ointment	16-fold greater kill; lesion score ↓ NR% vs. baseline
Rihova, M. (2025) [20]	Log_10_ CFU reduction on porcine-skin explant (24 h)	1 mg cm^−2^ fibre mat	Untreated control	3.1 log_10_ CFU drop
Tan, Y.Y. (2023) [21]	Zone of inhibition (ZOI)	16 mg mL^−1^ nanogel	Benzoyl-peroxide 5%	ZOI = 31 ± 2 mm vs. 28 ± 3 mm
Al-Momani, H. (2024) [14]	MIC (planktonic)/MBIC (biofilm)	MIC = 250 µg mL^−1^; MBIC = 62.5 µg mL^−1^	Green Ag NPs	ZnO equal MIC, superior MBIC
Raj, M. (2012) [22]	MIC	500 µg mL^−1^	Clindamycin 2 µg mL^−1^	ZnO less potent (MIC ratio 250:1)
Pati, R. (2014) [23]	Lesion bacterial load (CFU g^−1^ skin, day 7)	10 µg dose topically q12 h	Vehicle	↓ 2.4 log_10_ CFU
Zhang, F. (2025) [24]	Fibroblast intracellular Zn^2+^ (ICP-MS)	50 µg mL^−1^ ZnO + *C. acnes*	ZnO alone	+158% Zn^2+^ accumulation
Wang, S. (2024) [25]	Ear-swelling inhibition (% vs. control)	100 µg ZnO per ear	Dexamethasone 0.05%	62% vs. 70%

*C. acnes*, *Cutibacterium acnes*; *S. epidermidis*, *Staphylococcus epidermidis*; CFU, colony-forming units; log10, base-10 logarithm; MIC, minimum inhibitory concentration; MBIC (also written BIC in some studies), minimum biofilm inhibitory concentration; ICP-MS, inductively coupled plasma–mass spectrometry; Ag NPs, silver nanoparticles; HA-ZnO, hyaluronic acid–stabilized ZnO nanoparticles; q12 h, every 12 h; vs., versus; NR, not reported.

**Table 3:** Safety and mechanistic read-outs.

Study	Safety Endpoint	Key Numeric Finding	Mechanistic Note
Zhu, J. (2025) [19]	Zebrafish mortality (96 h)	0% at 100 µg mL^−1^	HA shell minimises free Zn^2+^
Rihova, M. (2025) [20]	Porcine-skin TEWL increase	+5% vs. baseline	Fibre acts as semi-occlusive barrier
Tan, Y.Y. (2023) [21]	Human patch-test erythema (% subjects)	0/20 volunteers	Plant metabolites may blunt ROS
Al-Momani, H. (2024) [14]	Cytotoxicity (HaCaT, MTT IC_50_)	312 µg mL^−1^	Ag–ZnO synergy allows lower dosing
Raj, M. (2012) [22]	Hemolysis at 1 mg mL^−1^	NR	NR
Pati, R. (2014) [23]	Histological inflammation score (0–4)	1.2 ± 0.3 vs. 3.8 ± 0.4 (control)	ROS burst plus membrane disruption
Zhang, F. (2025) [24]	Wound closure delay	+38% time to re-epithelialisation	Zn^2+^ suppresses MMP-9/TGF-β1 axis
Wang, S. (2024) [25]	Splenic T-cell proliferation	↓ 46% at 100 µg dose	ZnO skews Th1→Treg balance

TEWL, transepidermal water loss; HaCaT, human immortalized keratinocyte cell line; MTT, 3-(4,5-dimethylthiazol-2-yl)-2,5-diphenyltetrazolium bromide (viability assay); IC_50_, half-maximal inhibitory concentration; ROS, reactive oxygen species; Zn^2+^, divalent zinc ion; MMP-9, matrix metalloproteinase-9; TGF-β1, transforming growth factor-β1; Th1, T helper 1; Treg, regulatory T cell; CHS, contact hypersensitivity; NR, not reported.

**Table 4:** Physicochemical and dosing parameters.

Study	Primary Particle Size (nm)	Zeta Potential (mV)	ZnO Dose Tested	Exposure Duration	Release or Ionisation Data
Zhu, J. (2025) [19]	45 ± 8	−12.5	32 µg mL^−1^	24–72 h	Selective Zn^2+^ release ≤ 1.2 ppm
Rihova, M. (2025) [20]	28 ± 5	−18.0	1 mg cm^−2^ fibre	24 h	NR
Tan, Y.Y. (2023) [21]	20 nm (TEM)	−9.7	2–16 mg mL^−1^	48 h	NR
Al-Momani, H. (2024) [14]	50 ± 7	−22.4	15–250 µg mL^−1^	48 h	ZnO dissoln < 3%
Raj, M. (2012) [22]	~40	NR	0.1–1 mg mL^−1^	24 h	NR
Pati, R. (2014) [23]	35 ± 6	−10.2	10 µg topical	7 days	ICP: skin Zn 3.5 µg g^−1^
Zhang, F. (2025) [24]	20.3 ± 6.7	−8.38	12.5–50 µg mL^−1^	≤12 h cell; 7 d rat	Intracellular Zn^2+^ + 158%
Wang, S. (2024) [25]	30 nm	−15	100 µg per ear	72 h	NR

nm, nanometre; mV, millivolt; h, hour(s); d, day(s); ppm, parts per million; TEM, transmission electron microscopy; ICP, inductively coupled plasma; Zn^2+^, divalent zinc ion; NR, not reported.

## Data Availability

Not applicable.
